# Cellular redox state as a critical factor in initiating early embryonic-like program in embryonic stem cells

**DOI:** 10.1038/s41421-019-0127-5

**Published:** 2019-12-10

**Authors:** Chao Zhang, Yao-Long Yan, Jing Hao, Yangming Wang

**Affiliations:** 1 Academy for Advanced Interdisciplinary Studies, Peking University, Beijing, China; 2 Institute of Molecular Medicine, Peking University, Beijing, China

**Keywords:** Embryonic stem cells, Totipotent stem cells

Dear Editor,

Embryonic stem cells (ESCs) are pluripotent stem cells that can efficiently generate all embryonic but not extraembryonic tissues^1^. However, a small percentage (0.1–1%) of totipotent-like cells arise spontaneously in ESC cultures^2^, which have expanded cell fate potential to differentiate into both embryonic and extraembryonic cells. Intriguingly, these cells express high levels of transcripts including MERVL family of retroviruses and Zscan4 that are specifically activated in two-cell stage during embryo development^3^. For these reasons, these rare cells are also called totipotent-like cells or 2C-like cells. Furthermore, these cells can be labeled with a fluorescence protein reporter driven by the LTR of MERVL retroviruses^2^, for example, MERVL-LTR::tdTomato (2C::tdTomato) reporter. Currently, the molecular factors contributing to the emergence of 2C-like state are still not clear.

Zscan4 expression marks an intermediate state that precedes the 2C-like state^3^. To identify pathways that initiate the emergence of 2C-like state, we performed RNA-Seq with purified Zscan4::GFP-positive ESCs. Totally there were 721 and 882 genes upregulated and downregulated for more than two fold in Zscan4::GFP positive versus negative ESCs (Supplementary Table S1). Interestingly, KEGG pathway analysis identified glutathione metabolism significantly enriched in downregulated genes with a fold of enrichment as 4.2 and P value as 0.001 (Supplementary Fig. S1a). Gene set enrichment analysis (GSEA) confirmed the overall reduction of glutathione metabolism in Zscan4::GFP-positive ESCs (Supplementary Fig. S1b). Glutathione is one of the most important antioxidants in cells, and its metabolism is known to affect cellular redox state^4^. Based on this finding, we then checked whether the level of reactive oxygen species (ROS) is different between 2C-like cells and normal ESCs. Very strikingly, ROS level was significantly higher in 2C::tdTomato positive than negative cells (Fig. 1a, b), indicated by 2′, 7′-dichlorodihydrofluorescein diacetate (H2DCFDA), a chemically reduced form of fluorescein used as a general indicator for ROS in cells. In addition, a genetically coded fluorescent sensor HyPer^5^ showed that hydrogen peroxide level was significantly increased in 2C::tdTomato-positive cells (Supplementary Fig. S2). These results reveal an abnormal redox state characterized by increased ROS level in 2C-like cells that arise spontaneously in ESC culture.

**Fig. 1:**
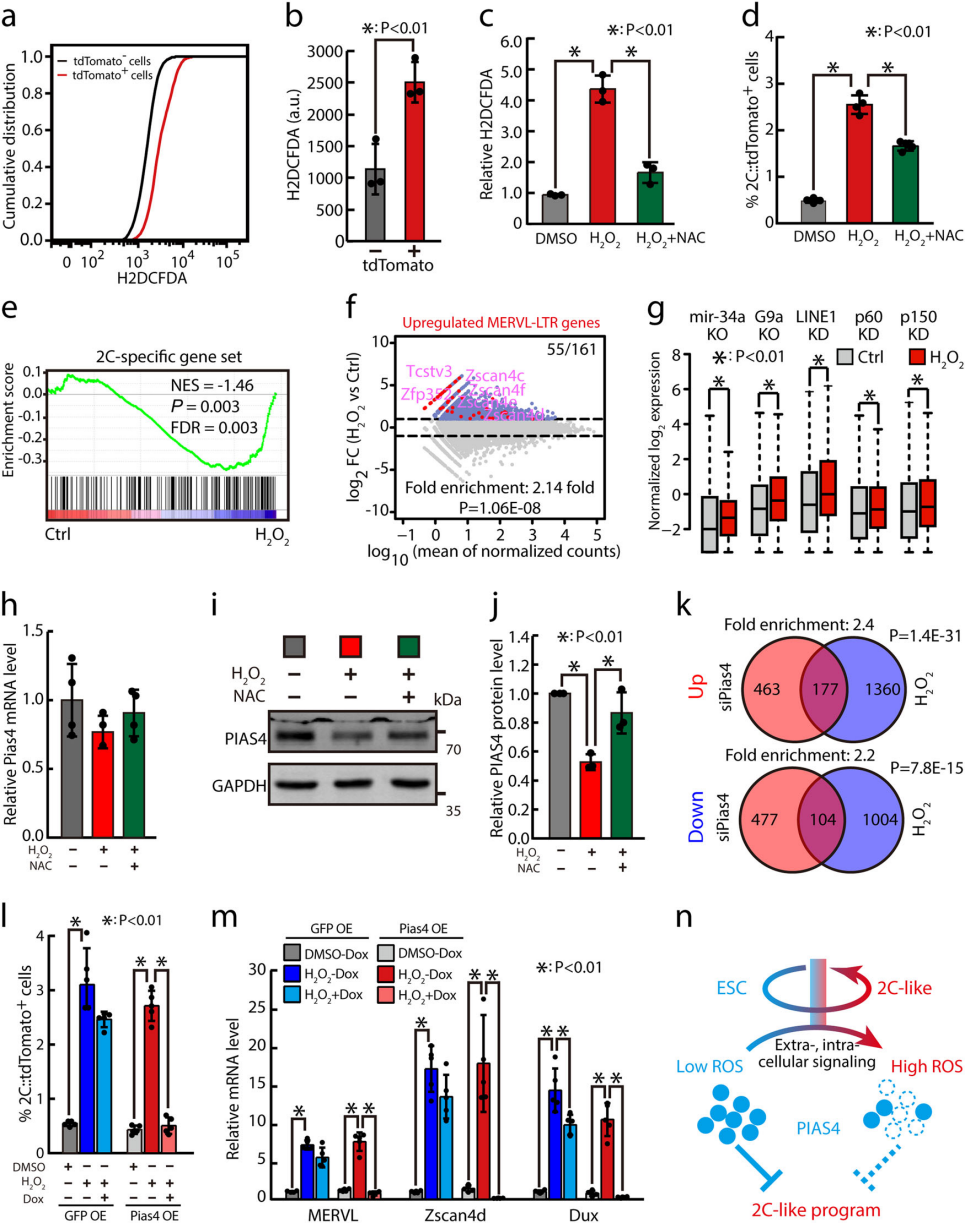
Cellular redox state regulates the activation of 2C-like program through regulating the stability of PIAS4 protein. **a** Representative flow cytometry analyses shown as cumulative distribution plot of H2DCFDA intensity in 2C::tdTomato positive and negative ESCs. **b** Quantification of mean H2DCFDA intensities (arbitrary unit) in 2C::tdTomato positive and negative ESCs. Mean ± SD are shown, *n* = 3. The *p* value was calculated by two-tailed Student’s *t* test. **c** Relative ROS level quantified by H2DCFDA staining after DMSO or H_2_O_2_ treatment with or without addition of NAC. Data were normalized to DMSO treatment. Mean ± SD are shown, *n* = 3. The *p* value was calculated by one-way ANOVA with two-tailed Dunnett’s test. **d** Fraction of 2C::tdTomato-positive cells after DMSO or H_2_O_2_ treatment with or without addition of NAC. Mean ± SD are shown, *n* = 4. The *p* value was calculated by one-way ANOVA with two-tailed Dunnett’s test. **e** GSEA for 2C-specific genes in untreated control or H_2_O_2_-treated ESCs. For the *x* axis, genes were ranked based on the ratio of untreated control versus H_2_O_2_-treated ESCs. **f** MA plots showing gene expression changes in H_2_O_2_-treated ESCs. Red dots indicate MERVL-LTR-driven genes. Out of 161 MERVL-LTR-driven genes, 55 were upregulated in H_2_O_2_-treated ESCs. Fold enrichment and *p* value are shown. The *p* value was calculated by hypergeometric test. **g** Box-and-whisker plots showing expression of genes upregulated by mir-34a knockout, G9a knockout, LINE1 knockdown, and Caf-1 p150 or p60 subunit knockdown in cells treated with H_2_O_2_. The *p* value was determined by Wilcoxon signed-rank test. **h** RT-qPCR of Pias4 mRNA in ESCs treated with H_2_O_2_ with or without addition of NAC. The β-actin gene was used as a control. Data were normalized to DMSO treatment. Mean ± SD are shown, *n* = 4. Sequences of qPCR primers are listed in Supplementary Table S3. **i** Representative western blotting image of PIAS4 protein in ESCs treated with H_2_O_2_ with or without addition of NAC. **j** Quantification of PIAS4 protein in ESCs treated with H_2_O_2_ with or without addition of NAC. Data were normalized to GAPDH and then to untreated ESCs. Mean ± SD are shown, *n* = 3. The *p* value was calculated by one-way ANOVA with two-tailed Dunnett’s test. **k** The Venn diagram (Up) shows the overlap between siPias4-upregulated and H_2_O_2_-upregulated genes, and the Venn diagram (Bottom) shows the overlap between siPias4-downregulated and H_2_O_2_-downregulated genes. Fold enrichment and *p* value are shown. The *p* value was calculated by hypergeometric test. **l** Fraction of 2C::tdTomato-positive cells in DMSO or H_2_O_2_-treated ESCs with or without Pias4 overexpression. Mean ± SD are shown, *n* = 5. The *p* value was calculated by one-way ANOVA with two-tailed Dunnett’s test. **m** RT-qPCR of MERVL, Zscan4d, and Dux in DMSO or H_2_O_2_-treated ESCs with or without Pias4 overexpression. The β-actin gene was used as a control. Data were normalized to DMSO-treated ESCs transfected with control overexpression vectors with no addition of doxycycline. Mean ± SD are shown, n = 5. The *p* value was calculated by one-way ANOVA with two-tailed Dunnett’s test. Sequences of qPCR primers are listed in Supplementary Table S3. **n** Summary graph. High ROS level destabilizes PIAS4 protein, in turn leading to the activation of 2C-like transcriptional program

To test whether high ROS level can cause the transition of ESCs into 2C-like state, we treated ESCs with hydrogen peroxide and found that the fraction of 2C::tdTomato-positive cells was indeed significantly increased by the treatment (Fig. 1c, d). Consistent with the causative role of ROS, addition of ROS scavenger N-acetyl-cysteine (NAC) significantly repressed the effect of hydrogen peroxide (Fig. 1c, d). To further support that hydrogen peroxide promotes the emergence of 2C-like state, we performed RNA-Seq analysis of hydrogen peroxide-treated ESCs (Supplementary Table S2). The results showed that hydrogen peroxide-treated ESCs significantly enriched 2C-specific ZGA transcripts (Fig. 1e). In addition, a significant fraction of MERVL-LTR-driven genes were also upregulated in hydrogen peroxide-treated ESCs (Fig. 1f). Previously, knocking out miR-34a^6^ or G9a^2^ and knocking down LINE1^7^ or CAF-1 (p150 and p60)^8^ have been shown to activate 2C-like program. Consistently, genes upregulated in these conditions were also significantly induced by hydrogen peroxide (Fig. 1g). Finally, hydrogen peroxide also triggered 2C-like program in E14 cells and 2C::tdTomato R1 cells, which was significantly rescued by the addition of ROS scavenger NAC (Supplementary Fig. S2d, e). These results suggest that increased hydrogen peroxide activates 2C-like program in mouse ESCs.

We then tested whether ROS-inducing small molecules can also promote the activation of 2C-like state. Camptothecin (CPT), zeocin, and azidothymidine (AZT) significantly increased ROS production in ESCs (Supplementary Fig. S3a). Consistently, these molecules also significantly increased the fraction of 2C::tdTomato-positive cells (Supplementary Fig. S3b). In addition, ROS scavenger NAC repressed their effects on the induction of 2C-like cells (Supplementary Fig. S3a, b). qPCR analysis confirmed that these small molecules upregulated 2C-specific transcripts MERVL and Zscan4 through increasing ROS in ESCs (Supplementary Fig. S3c, d). Diphenyleneiodonium (DPI) is an inhibitor for NADPH oxidases and Dual oxidases that produce ROS in mammalian cells^9^. Interestingly, adding DPI into ESC culture significantly decreased the overall ROS level and the percentage of 2C::tdTomato-positive cells (Supplementary Fig. S3e, f). These results suggest that small molecules affecting ROS production may be exploited to activate or repress 2C-like program in ESCs.

Recently, we identified a Sumo2 E3 ligase PIAS4 as a regulator of 2C-like state, whose protein but not mRNA is significantly diminished in 2C-like cells^10^. We checked whether hydrogen peroxide can modulate PIAS4 protein level in ESCs. Interestingly, hydrogen peroxide treatment led to a significant decrease of PIAS4 protein, but had little effect on Pias4 mRNA (Fig. 1h–j). Moreover, proteasome inhibitor MG132 rescued the protein level of PIAS4 upon hydrogen peroxide treatment (Supplementary Fig. S4a, b), suggesting that hydrogen peroxide decreases the stability of PIAS4 protein. Intriguingly, RNA-Seq analysis showed significant overlaps between genes changed by hydrogen peroxide treatment and genes changed by Pias4 knocking down (Fig. 1k), although the number of genes affected by hydrogen peroxide was almost as twice as the number of genes affected by Pias4 knocking down. These data suggest that hydrogen peroxide activates 2C-like program at least partially through destabilizing PIAS4.

To further support that PIAS4 acts downstream of hydrogen peroxide, we constructed doxycycline-inducible Pias4-overexpressing ESCs. Consistently, PIAS4 overexpression blocked the increase of 2C-like cell populations upon hydrogen peroxide treatment (Fig. 1l; Supplementary Fig. S4c). RT-qPCR also confirmed that Pias4 overexpression blocked the increase of 2C-specific transcripts including MERVL, Zscan4d, and Dux (Fig. 1m). Moreover, Pias4 knocking down led to the increase of 2C-like cells with no alteration of cellular ROS level (Supplementary Fig. S4d, e), and NAC did not block the increase of 2C-like cells by Pias4 knocking down (Supplementary Fig. S4f, g). These data are consistent with PIAS4 protein functioning downstream, but not upstream of hydrogen peroxide. Together, these results suggest that high ROS level can trigger the generation of 2C-like state through the destabilization of PIAS4 protein.

Collectively, our study identified cellular redox state as a key factor regulating the cycling of 2C-like state in ESCs, and that PIAS4 may act downstream of ROS signaling to orchestrate the initiation of early embryonic-like program in ESCs (Fig. 1n). Future studies should identify the upstream factors that cause the shift of redox state in ESCs during the initiation of 2C-like program and components of the redox signaling cascade that eventually shape the epigenetic program in ESCs. In addition, 2C-like cells reactivate numerous genes specifically expressed during zygotic genome activation (ZGA)^2^; our study raises a possibility that ROS signaling may play a role during ZGA.

## Supplementary information

Supplementary Information

Supplementary Tables S1-3

## Supplementary information

**Supplementary Information** accompanies the paper at (10.1038/s41421-019-0127-5).
