# Supplementary material for: Cellular redox state as a critical factor in initiating early embryonic-like program in embryonic stem cells
Source: Cell Discov. 2019 Dec 10;5:59. doi: 10.1038/s41421-019-0127-5 (PMC6901566; doi:10.1038/s41421-019-0127-5)
Supplement: Supplementary file 1 — Supplementary Information [file 41421_2019_127_MOESM1_ESM.pdf]

# Supplementary Information

## Cellular redox state as a critical factor in initiating early embryonic-like program in embryonic stem cells

**Authors:** Chao Zhang[2#], Yao-Long Yan[1#], Jing Hao[1#], Yangming Wang[1,*]

**Affiliations:**

1 Institute of Molecular Medicine, Peking University, Beijing, China

2 Academy for Advanced Interdisciplinary Studies, Peking University, Beijing, China

#These authors contributed equally to this study.

*Correspondence to: yangming.wang@pku.edu.cn (YW)

**Supplementary Figure S1-4 with Figure Legends**

**Title and Legends for Supplementary Table S1-3**

**Supplementary Materials and Methods**

**Supplementary Figure S1**

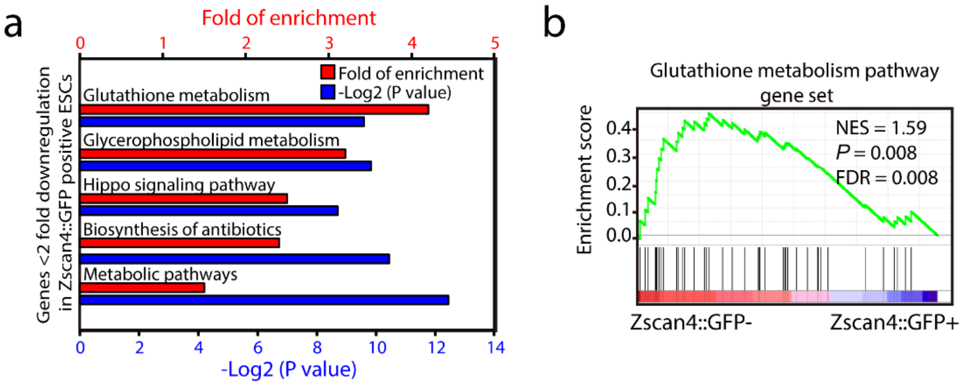


**Supplementary Figure S1. Genes in glutathione metabolism pathway are downregulated in Zscan4::GFP positive ESCs.**

**a)** KEGG pathway analysis of downregulated genes in Zscan4::GFP positive cells. Top 5 enriched pathways are shown with P values and fold of enrichment.

**b)** GSEA for glutathione metabolism pathway related genes in Zscan4::GFP negative versus positive cells. For x-axis, genes were ranked based on the ratio of Zscan4::GFP negative versus Zscan4::GFP positive cells.

**Supplementary Figure S2**

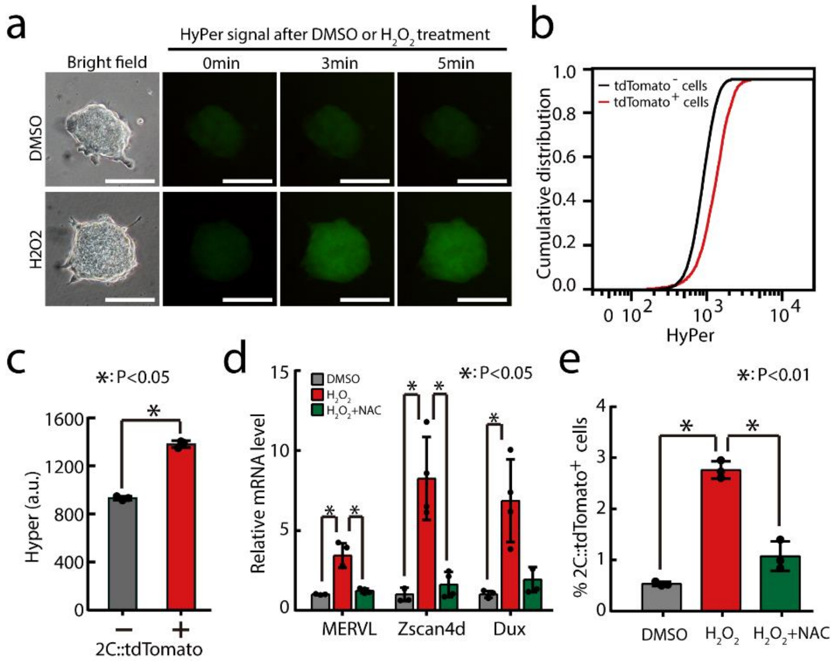


**Supplementary Figure S2. Genetically encoded ROS sensor HyPer detected higher ROS level in 2C::tdTomato positive than negative ESCs.**

**a)** Assay to verify genetically encoded HyPer as a ROS sensor in ESCs. HyPer fluorescence intensity increases after $H_2O_2$ but not DMSO treatment. Representative images of an ESC colony are shown. Scale bars, 50 µm.

**b)** Representative flow cytometry analyses shown as cumulative distribution plot of HyPer intensity in 2C::tdTomato positive and negative ESCs.

**c)** Quantification of mean HyPer intensity in 2C::tdTomato positive and negative ESCs. Shown are mean ± SD, n = 3. The *p*-value was calculated by two-tailed Student's *t* test.

**d)** RT-qPCR of MERVL, Zscan4d and Dux in DMSO or H2O2 treated E14 ESCs with or without addition of NAC. The β-actin gene was used as a control. Data were normalized to DMSO treatment. Shown are mean ± SD, n = 4.

**e)** Fraction of 2C::tdTomato positive cells after DMSO or H2O2 treatment with or without addition of NAC in 2C::tdTomato reporter R1 ESCs. Shown are mean ± SD, n = 4. The p-value was calculated by one-way ANOVA with two-tailed Dunnett's test.

**Supplementary Figure S3**

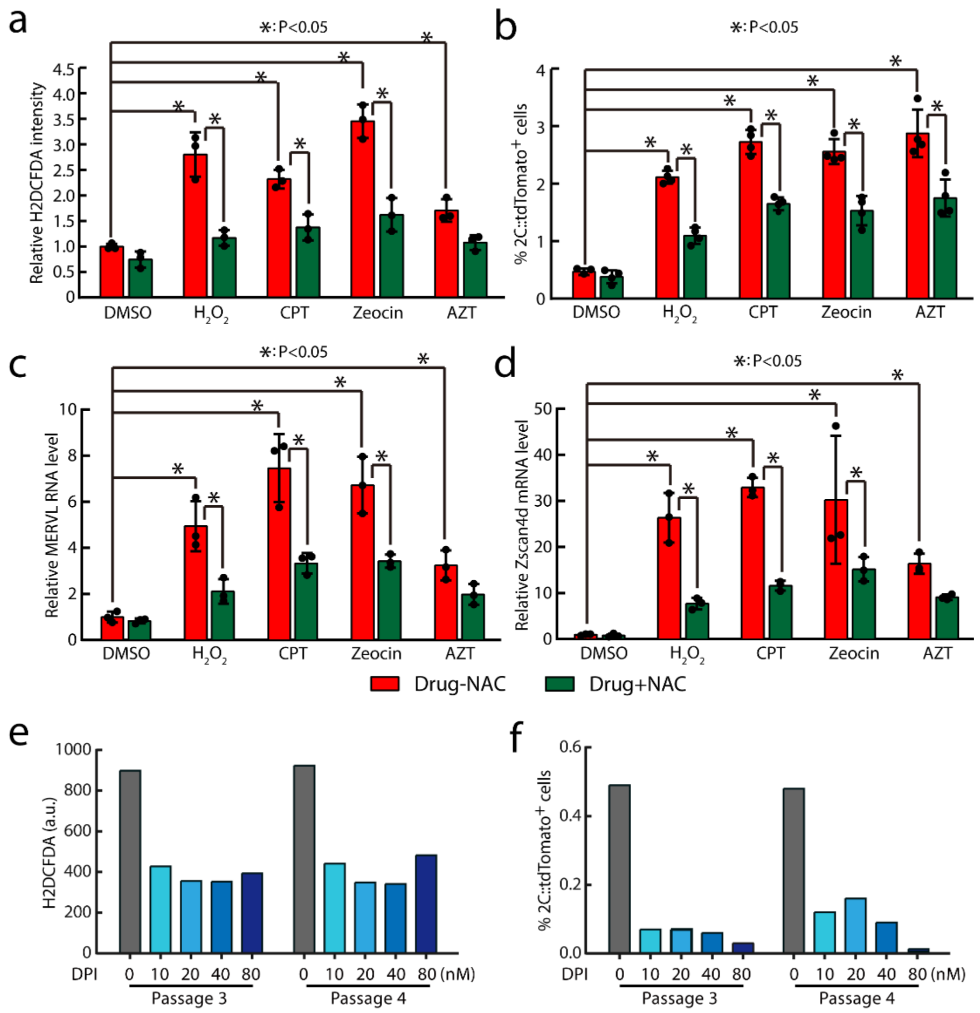


**Supplementary Figure S3. Various small molecules change the percentage of 2C-like cells through modulating ROS level in ESC culture.**

**a)** Relative ROS level indicated by H2DCFDA staining after DMSO, $H_2O_2$, CPT, zeocin or AZT treatment with or without addition of NAC. Data were normalized to DMSO treatment without addition of NAC. Shown are mean ± SD, n = 3. The *p*-value was calculated by one-way ANOVA followed by two-tailed Dunnett's test.

**b)** Fraction of 2C::tdTomato positive cells after DMSO, $H_2O_2$, CPT, zeocin or AZT treatment with or without addition of NAC. Shown are mean ± SD, n = 4. The *p*-value was calculated by one-way ANOVA followed by two-tailed Dunnett's test.

RT-qPCR of **c)** MERVL and **d)** Zscan4d after DMSO, $H_2O_2$, CPT, zeocin or AZT treatment with or without addition of NAC. The β-actin gene was used as a control. Data were normalized to DMSO treatment without addition of NAC. Shown are mean ± SD, n = 3. The *p*-value was calculated by one-way ANOVA with two-tailed Dunnett's test.

**e)** ROS level quantified by H2DCFDA staining in ESCs treated with DPI for 3 or 4 passages at 10, 20, 40 and 80 nM concentrations.

**f)** Fraction of 2C::tdTomato positive cells in ESCs treated with DPI for 3 or 4 passages at 10, 20, 40 and 80 nM concentrations.

**Supplementary Figure S4**

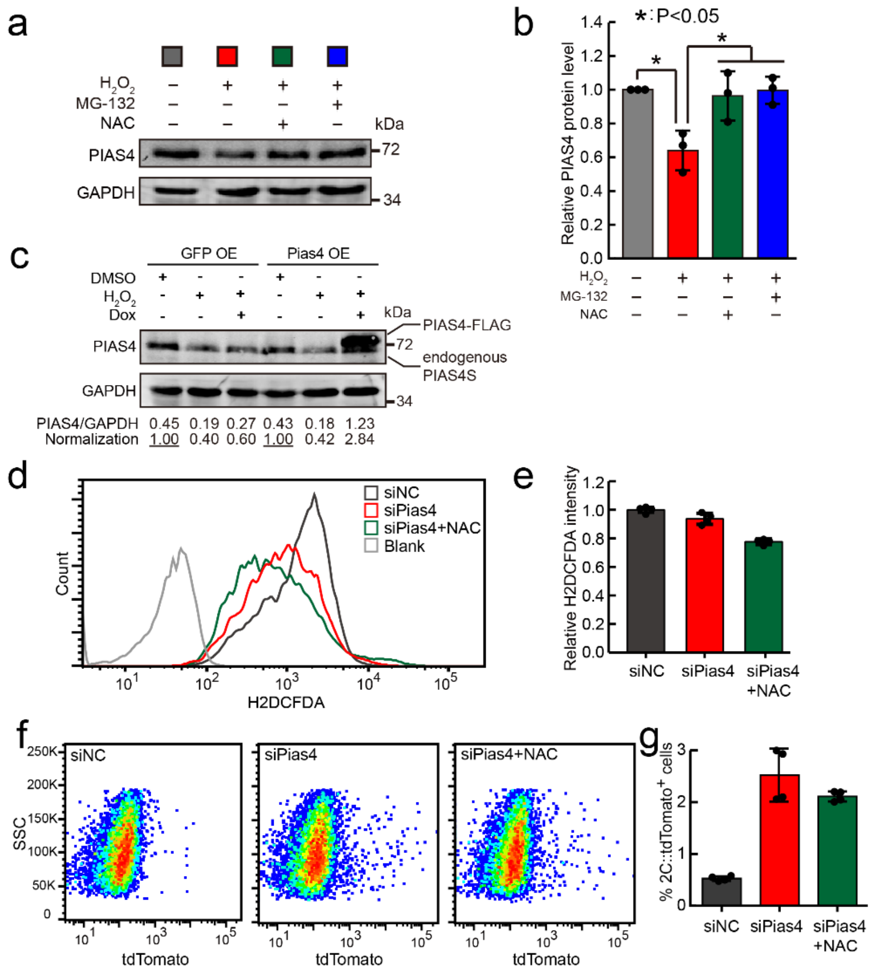


**Supplementary Figure S4. Hydrogen peroxide induces the emergence of 2C-like cells through destabilizing PIAS4 protein.**

**a)** Representative western blotting image for PIAS4 protein in ESCs after $H_2O_2$ treatment with or without addition of NAC or proteasome inhibitor MG132.

**b)** Quantification of PIAS4 protein from western blotting analyses. Data were normalized to GAPDH and then to untreated ESCs. Shown are mean ± SD, n = 3. The *p*-value was calculated by one-way ANOVA with two-tailed Dunnett's test.

**c)** Representative western blotting image for PIAS4 protein in DMSO or $H_2O_2$ treated ESCs with or without Pias4 overexpression.

**d)** Representative flow cytometry analyses shown as histograms of H2DCFDA intensity in siNC or siPias4 treated ESCs with or without addition of NAC.

**e)** Quantification of mean H2DCFDA intensity from flow cytometry analyses. Data were normalized to ESCs treated with siNC without addition of NAC. Shown are mean ± SD, n = 4.

**f)** Representative flow cytometry analyses shown as dot plots for 2C::tdTomato fluorescence intensity in siNC or siPias4 treated ESCs with or without addition of NAC.

**g)** Quantification of fraction of 2C::tdTomato positive cells in siNC or siPias4 treated ESCs with or without addition of NAC. Shown are mean ± SD, n = 4.

## Title and Legends for Supplementary Table S1-3

**Supplementary Table S1.** FPKM of genes in Zscan4::GFP negative and positive ESCs; Shown is the average of two replicates.

**Supplementary Table S2.** FPKM of genes in untreated control and hydrogen peroxide treated ESCs; Shown is the average of two replicates.

**Supplementary Table S3.** Sequences for real time qPCR primers

## Supplementary Materials and Methods

### Cell culture and construct of reporter cell lines

Embryonic stem cells were grown in KnockOut™ DMEM (Gibco, Cat. # 10829081) supplemented with 15% FBS (Hyclone, Cat. # SH3007103), 1,000 U/ml mouse leukemia inhibitory factor (1,000 U/ml), 0.1 mM non-essential amino acids (Gibco, Cat. # 11140050), 1 mM L-glutamine (Gibco, Cat. # 25030081), 0.1 mM β-mercaptoethanol, and penicillin (100 U/ml) and streptomycin (100 μg/ml). Zscan4::EGFP and 2C::tdTomato ESCs were made as previously described[1-3]. For hydrogen peroxide experiment, typically 200 μM hydrogen peroxide was used in cell culture. N-acetylcysteine (Sigma, Cat. # A9165) was used at 1 mM concentration. 100 nM Camptothecin, 12.5 μg/ml zeocin and 10 μM azidothymidine were used to induce ROS production. Diphenyleneiodonium was used to inhibit the action of NOX with a gradient concentration of 10nM, 20nM, 40nM and 80nM. Mycoplasma detection tests were conducted routinely to ensure mycoplasma free conditions throughout the study.

### RNA extraction and RT-qPCR

Total RNA was extracted from cells with Trizol reagent (Invitrogen, Cat. # 15596026). For quantitative PCR with reverse transcription (RT-qPCR) analysis, cDNA were obtained from 500 ng total RNA using HiScript II Q RT SuperMix for qPCR (Vazyme, Cat. # R223). qPCR assay were carried out with AceQ qPCR SYBR Green Mater Mix reagent (Vazyme, Cat. # Q141) in 96-well dishes in three biological replicates on StepOne Plus Real-Time PCR System (Applied Biosystems) with standard protocols.

The expression levels were standardized by $\beta$-actin housekeeping gene expression. Primers for qPCR are listed in Supplementary Table S3.

**Immunoblots**

Cells were harvested and lysed in Mammalian Protein Extraction Reagent (Cwbiotech, Cat. # CW0889) with Protease Inhibitor Cocktail (100x, Cwbiotech, Cat. # CW0889). Proteins were quantified using Pierce™ BCA Protein Assay Kit (Thermo Scientific). Equal amounts of proteins were loaded for immunoblotting. Antibodies used: rabbit anti-PIAS4 (1:1,000, Proteintech, Cat. # 14242-1-AP), rabbit anti-GAPDH (1:1,000, Bioworld Technology, Cat. # MB001). Anti-rabbit secondary antibodies were from LI-COR and membranes were imaged using Oddssey.

**Flow cytometry**

Cells were collected in ice-cold PBS containing 2% FBS. Cell sorting was performed on BD FACSAria III. During sorting, cells were collected in ESC culture medium at 4℃. Quantification of the population of 2C::tdTomato positive cells and Zscan4::GFP positive cells were performed by BD LSRFortessa SORP. Data were analyzed using FlowJo software.

**ROS level quantification**

The probe H2DCFDA (Molecular Probes, Cat. # C6827) was used for ROS measurement according to the manufacturer's instructions with slight modification. In brief, cells were collected and resuspended in pre-warmed HBSS buffer (Gibco™, Cat. # 14025092) containing 1 μM H2DCFDA. Incubate at 37℃ for 20 minutes and wash cells gently three times with warm PBS to remove unloaded probe. Then analyze the

cells by flow cytometry. The quantification was plotted by the mean fluorescence intensity or cumulative distribution function. For HyPer assay, HyPer sequence was cloned into PiggyBac plasmid and stably transfected into ESCs containing 2C::tdTomato reporter. Then the cells were selected with hygromycin for 5 d. Colonies were then picked and expanded. Single wavelength (500 nm) was monitored for HyPer signal.

**siRNA transfection**

ESCs were transfected with indicated siRNAs using DharmaFECT 1 (Dharmacon, Cat. # T-2001) reagent following the standard procedure. The sequence of siPias4 is 5'-CAACAAGCCUGGUGUGGAA-3'. NC is a siRNA with the sequence derived from *C. elegans* genome that does not target any mammalian genes, provided by GenePharma (Shanghai) company as a control for siRNA transfection. For analysis of flow-cytometry, cells were collected at 48hrs after transfection.

**RNA-seq and bioinformatics analysis**

Total RNA was purified by poly-T oligo-attached magnetic beads twice and then were used to generate double-stranded (ds) cDNA. The ds-cDNA was ligated to adaptors and sequenced by Illumina Genome Analyzer (Novogene). Reads were aligned to the mouse genome (mm10) with STAR (version 2.5.0) using the GENCODE transcript annotation as transcriptome guide. All programs were perform under the default settings except for special statements. Expression levels were quantified as normalized FPKM using Cufflinks (version 2.2.1). GSEA was used to calculate the enrichment of selected gene sets by java GSEA Desktop Application. R 3.5.1 were used for the

generation of MA plot, scatter plot, box plot and venn diagram. The 2C-specific ZGA genes are genes activated during ZGA (the 2C stage) that are also enriched in 2C::tdTomato$^{+}$ cells from ref.[4]. The list of MERVL-LTR driven transcripts was from ref.[5]. The list of genes induced by mir-34a knockout was from ref.[6]. The list of genes induced by G9a knockout was from ref.[4]. The list of genes induced by LINE1 knocking down was from ref.[7]. The lists of genes induced by p150 or p60 knocking down were from ref.[1]. The list of genes up- or down-regulated in siPias4 treated ESCs were from ref.[3].

**Quantification and statistical analysis**

The number of independent experimental replications, the definition of center and precisions measures are reported in the figure legends (n, mean ± SD). $p<0.05$ is generally considered as statistically significant. Statistical analyses were performed using the GraphPad Prism v6 software and R programming. Statistical significance was assessed by two-tailed t test except when specified in the figure legends. For boxplots, upper and lower whiskers are defined as respectively Q3 + 1.5 x IQR and Q1 - 1.5 x IQR with Q1 and Q3 being the first and third quartile of the plotted distribution and IQR the inter-quartile range, and the p-value was determined by Wilcoxon signed rank test. For multiple comparison, the p-value was calculated by one-way or two-way ANOVA followed with Dunnett's test. And hypergeometric test was used for venn diagram significant test.

**Data and materials availability**

All data generated or analysed during this study are included in the manuscript and its supplementary information files. Original sequencing data used and/or analysed during the current study are available from the corresponding author on reasonable request.

## References


1 Ishiuchi, T. *et al.* Early embryonic-like cells are induced by downregulating replication-dependent chromatin assembly. *Nature structural & molecular biology* **22**, 662-671, doi:10.1038/nsmb.3066 (2015).

2 Rodriguez-Terrones, D. *et al.* A molecular roadmap for the emergence of early-embryonic-like cells in culture. *Nature genetics* **50**, 106-119, doi:10.1038/s41588-017-0016-5 (2018).

3 Yan, Y. L. *et al.* DPPA2/4 and SUMO E3 ligase PIAS4 opposingly regulate zygotic transcriptional program. *PLoS biology* **17**, e3000324, doi:10.1371/journal.pbio.3000324 (2019).

4 Macfarlan, T. S. *et al.* Embryonic stem cell potency fluctuates with endogenous retrovirus activity. *Nature* **487**, 57-63, doi:10.1038/nature11244 (2012).

5 Eckersley-Maslin, M. A. *et al.* MERVL/Zscan4 Network Activation Results in Transient Genome-wide DNA Demethylation of mESCs. *Cell Rep* **17**, 179-192, doi:10.1016/j.celrep.2016.08.087 (2016).

6 Choi, Y. J. *et al.* Deficiency of microRNA miR-34a expands cell fate potential in pluripotent stem cells. *Science* **355**, doi:10.1126/science.aag1927 (2017).

7 Percharde, M. *et al.* A LINE1-Nucleolin Partnership Regulates Early Development and ESC Identity. *Cell* **174**, 391-405 e319, doi:10.1016/j.cell.2018.05.043 (2018).
